# HBV and HCV Coinfection among HIV/AIDS Patients in the National Hospital of Tropical Diseases, Vietnam

**DOI:** 10.1155/2014/581021

**Published:** 2014-12-08

**Authors:** Bùi Vũ Huy, Kanxay Vernavong, Nguyễn Văn Kính

**Affiliations:** ^1^Hanoi Medical University, Hanoi 10000, Vietnam; ^2^National Hospital of Tropical Diseases, 78 Giai Phong Road, Dong Da District, Hanoi 10000, Vietnam

## Abstract

*Aim*. To examine prevalence and characterization of HBV and HCV coinfection among HIV/AIDS patients. *Methods*. This cross-sectional, retrospective study analyzed 724 HIV/AIDS patients in the HIV clinic at the National Hospital of Tropical Diseases (NHTD), from 5/2005 to 4/2011. *Results*. The prevalence of HBV, HCV, and HIV coinfection was 50.3% (364/724), of which HbsAg, HCV, and both of HbsAg, and HCV positivity were 8.4%, 35.4%, and 6.5%, respectively. The cohort (364 patients) with HBV, HCV, and HIV coinfection live in the 30 provinces/cities in the North and Central area of Vietnam. We found statistically significant associations between heightened risk of coinfection with HIV and HCV in the age group 30–39 years (*P* < 0.001), male gender (*P* < 0.001), never married patients (*P* < 0.001), patients with a history of injection drug use (*P* < 0.001), and clinical stages 2–4 (*P* < 0.001). Coinfection with HBV/HIV was statistically significant associations between heightened risk of marital status (never married) (*P* < 0.001) and those who reported transmission through sexual intercourse. *Conclusion*. Coinfection with viral hepatitis is common in HIV patients; further study of the impact and evolution of coinfection is necessary to find effective treatment algorithms.

## 1. Introduction

Hepatitis B virus (HBV) and hepatitis C Virus (HCV) are among the primary causes of morbidity and mortality in HIV patients. Coinfection of HBV and HCV with HIV has been associated with reduced survival, increased risk of progression to liver disease, and increased risk of hepatotoxicity, associated with antiretroviral therapy [1–3].

HBV and HCV share common pathways of transmission with HIV, including injection drug use, sexual intercourse, and mother-to-child transmission [3, 4]. In the past few years, several guidelines and reviews have highlighted this problem and have provided recommendations about how to best manage patients coinfected with HIV and HBV, HCV [3–6].

However, studies showed that the prevalence of HBV, HCV, and HIV coinfection differs both by geographic region and according to behaviour of infected people [3, 4, 7, 8].

In Vietnam, previous studies have reported various prevalence values of HBV, HCV, and HIV coinfection. These studies were evaluated in different provinces and in individuals with different risk factors [9, 10], even assessed on blood samples stored [11]. According to these reports, in Bac Ninh province the HIV, HBV, and HCV prevalence was 42.4%, 80.9%, and 74.1% in injection drug user [9]; in Hai Phong city the cumulative HBV incidence rate was 53.2% in drug users, 51.6% in female sex workers, 54.3% in seafarers, and 50.5% in pregnant women [10].

National Hospital of Tropical Diseases (NHTD) is located in Hanoi, that is, a center hospital for infectious diseases in Viet Nam, including HIV/AIDS. Hospitalization patients come from regions of Vietnam, mostly from the Northern and the Central area of Vietnam. HIV/AIDS patients have been managing since the year 2003 at the outpatient clinic. These patients are being cared for and treated according to guidelines of WHO, including diagnosis, treatment (HIV, opportunity infection, and related diseases), and prophylaxis [3].

To examine the prevalence of hepatitis virus in HIV patients, this analysis aimed to assess the seroprevalence of HBsAg and HCV seropositivity within the HIV positive population in an HIV outpatient clinic at the NHTD and to identify demographic factors associated with HBV and HCV coinfection.

## 2. Methods

### 2.1. Study Design

This cross-sectional retrospective study aims to evaluate the prevalence of serological markers for HBV and HCV infections.

### 2.2. Studied Population

In the present study, all adult patients with HIV positive who have been managed in outpatient clinic of NHTD were analysed.

The sample included all adult patients aged 18 years or older attending for care and treatment HIV/AIDS during the period from 5/2005 to 4/2011, and patients who received highly active antiretroviral therapy (HAART) were included.

### 2.3. Data Collection

All of HIV patients were exploiting demographic assessment of clinical signs, opportunity infections, clinical stage classification, screening HBV, HCV, tuberculosis, and counted CD4 when they registered at outpatient clinic. The follow-up visits were done every month and CD4 count was repeated every 6 months. Patients were started on HAART based on CD4 count and clinical stage, in accordance with WHO guidelines [3].

In the study, data on patient demographics and features of HBV and HCV coinfection were collected and analyzed. Data collected on patients included sex, age, reported HIV transmission, HBV and HCV status, and AIDS-defining illnesses.

*Definitions*. (i) HBV/HIV coinfection was defined by a positive HBV surface antigen (HBsAg).

(ii) HCV/HIV coinfection was defined by a positive HCV antibody.

(iii) Prevalence of HBV coinfection, HCV coinfection, and HBV/HCV coinfection was calculated for those with recorded test results for HBsAg and HCV antibody.

Risk factors for coinfection with HBV and HCV were determined in cross-sectional analyses using Student's *t*-test for trend. Comparisons of proportions were analyzed using Chi-square tests. Significance was set at *P* < 0.05.

Stata software version 12.1 was used for all statistical analyses.

This study was approved by the Ethics Committee in Human Research at the NHTD.

## 3. Results

We reviewed data on 724 HIV positive patients treated at the HIV outpatient clinic in NHTD during the study period. Of these, 364 (50.3%) were determined to be coinfected. Of these, 61/724 were seropositive for only HBV (8.4%), 256/724 were seropositive for only HCV (35.4%), and 47 were seropositive for both HCV and HBV (6.5%).

The demographics, risk behaviors, and clinical stage at baseline of HIV positive population coinfection with HBV and HCV are shown in Table 1.

### 3.1. Age

There was a significant relationship between age and HCV coinfection rate with the group aged 30–39 years which is at higher risk than other age groups (*P* < 0.001). Finally, HBV coinfection and combined HBV and HCV coinfection in HIV positive patients were not statistically significant with respect to age group.

### 3.2. Gender

Regarding gender, there was a significant association (*P* < 0.001) between HCV only coinfection in HIV positive men compared with HIV positive women (50.3% and 8.5%, resp.); there were no differences between genders in rates of coinfection with HBV in HIV positive patients (7.3% and 10.4%, resp.). Finally, HBV/HCV coinfections in HIV positive men were higher than the HIV positive women (9.5% and 1.2%, resp.).

### 3.3. Marital Status

Marital status and HBV, HCV, and HIV coinfections status were significantly associated. The rates of HBV and HCV coinfection in HIV positive patients were higher among unmarried people than married people.

### 3.4. Geographic Domicile

The vast majority of HIV positive patients lived in Hanoi (40.9% [296/724]). Among the 724 HIV positive patients at outpatient clinic, the cohort (364 patients) with HBV, HCV, and HIV coinfection lived in the 30 provinces and cities in the North and Central area of Vietnam, and one-third live in the city of Hanoi (125/364).

### 3.5. Risk Factors Associated with Transmission Behaviours

A significant relationship was found between exposure to transmission risk factors and coinfection with either HCV or HBV (*P* < 0.001). In the HCV and HIV coinfection group, injection drug use had the strongest association (44% versus 28% and 12.2% resp.). Risk for HBV coinfection was the highest in the group identified as transmission by sexual intercourse (26% and 4%, resp.). However, risk for HBV/HCV coinfection in HIV positive patients was significantly associated with behaviors combining injection drug use and sexual intercourse.

### 3.6. Clinical Stage Classification

Based on clinical staging, the rate of HBV coinfection was not different in those with clinical stage. But the rates of HCV/HIV coinfections and HBV/HCV/HIV coinfection were higher in clinical stages 2 and 3 (*P* < 0.001).

## 4. Discussion

### 4.1. Prevalence of Hepatitis B and C Viruses and HIV Co-Infection

Overall, the prevalence of hepatitis B, hepatitis C, or both, with HIV coinfection, is 50.3%. Among these, HBV/HIV is 8.4%, HCV/HIV is 35.4%, and HBV/HCV/HIV is 6.5%. We did not assess prevalence of other hepatitis viruses in the HIV positive population, such as hepatitis A, hepatitis D, and hepatitis E. Varied prevalence rates of hepatitis and HIV coinfection have been reported by study by country, as well as by hepatitis subtype [7, 8, 10]. Overall, the prevalence of HCV/HIV is considerably higher than HBV/HIV and it is higher than those in industrialized countries [12]. With respect to HCV coinfection, our rates differ from some cross-sectional studies in Vietnam which have reported the prevalence of HCV/HIV that ranges from 74% [11] to 100% [9], findings that are similar to those reported from China which range from 62.4 to 93.6% in HIV-positive intravenous drug users [13]. These studies contrast with the very low prevalence reported in a study from India of 2%, where the predominant mode of acquiring HIV infection was 80% heterosexual contact, 6% transfusion of blood products, and 2.43% intravenous drug use [7]. It also varies from reports from an industrialized country, such as Australia (13.1%) [12]. However, the prevalence of HBV in our study is similar to other studies in Vietnam [11], in developing countries [7], and in industrialized countries [12].

We hypothesize that differences seen in the prevalence of different strains of hepatitis viruses relate to the population studied, transmission risk behaviours of the population, and the impact of harm reduction activities [3, 10]. A case-control study on risks for HIV, HBV, and HCV infections among male injection drug users in northern Vietnam showed that 64% of these IDUs share needles, drug solutions, containers, rinse water, and frontloading drugs [9]. Among them, only 12% reported never having had sexual intercourse and only half of the male IDUs reported using a condom at their last sexual intercourse. The model was significantly associated with HIV infection (OR = 2.8), HBV infection (OR = 3.8), and HCV infection (OR = 4.6). In another report in Vietnam, the authors showed that the prevalence of hepatitis B (HBsAg) ranged from 5.7% to 24.7% and anti-HCV ranged from 0.38% to 4.3% in the general population, while anti-HCV among IDUs ranged from 31% to 97.2%. The HBV prevalence among HIV population was similar to the general population, while HCV/HIV coinfection was concentrated in some groups and it can be as high as 98.5% among HIV-infected patients [14].

Prevention of hepatitis B and hepatitis C infections among HIV-infected patients in Vietnam has been of interest to public health authorities. No surveillance system is in place to monitor infection levels. No program exists to provide HIV persons with information on viral hepatitis infections and how to prevent them, nor are intervention programs available to provide necessary skills for avoiding infections. Standard needle and syringe exchange programs have been shown to be effective in preventing HIV transmission, but findings on these programs' effectiveness in HCV prevention are inconclusive.

### 4.2. Demographic Characteristic of HBV, HCV, and HIV Coinfected People

In this study, rates of HBV/HIV coinfection and HBV/HCV/HIV coinfection did not differ significantly in the age groups. However, rate of coinfection was significantly higher in the group aged 30–39 years (*P* < 0.001) in the HCV/HIV coinfection group. Here, too, the rate of hepatitis and HIV coinfection across different age groups has been reported to be widely different across studies and countries. As we found, reported rates of coinfection are considerably higher in the age group of 30–40 years in some studies from developing countries [7], while rates in industrialized countries were the highest in the age group aged over 40 years [12]. We hypothesize that the decline in rates of hepatitis/HIV coinfection in the age group over 40 years may be related to increased loss to follow-up, caused by the end-stage liver disease complication [6]. This should be of interest because it may be the reason for the limited HAART for the HIV positive population. This hypothesis should be investigated in a future study.

Independent risk behaviour for HBV and HCV coinfection was found in our study. As is known, both HBV infection and HCV infection share transmission routes, in particular parenteral exposure. HCV/HIV coinfection is more closely associated with injection drug use (*P* < 0.001), while HBV/HIV coinfection is more closely associated with sexual intercourse (*P* < 0.001). In addition, HCV/HIV and HBV/HCV/HIV coinfection are significantly higher in males than females (*P* < 0.001), but we do not see a statistically significant difference between sexes in HBV/HIV coinfection. IDUs in Vietnam were reported sharing needles, drug solutions, containers, and rinse water and have been significantly associated with HIV infection, HBV infection, and HCV infection in a prior study [9]. Prior research in Vietnam suggests that HIV transmission routes are highest from injection drug use [10, 11] particularly in men [11]. Demographic studies of hepatitis and HIV coinfection also showed that HCV/HIV coinfection was more common in injection drug users and more common in men than women [12]. Our study of this outpatient clinic population showed that most HIV positive patients have families (75%) and common domicile for patients with hepatitis and HIV coinfection is Hanoi (40.9%). However, group patients with hepatitis and HIV coinfection distribute in 30/63 provinces/cities of Vietnam and may be the source of infection in the community. Taken together, these factors suggest that this urban-dwelling, family-connected IDU population is a good target for an intervention strategy to prevent coinfection or transmission of either hepatitis or HIV.

Limitations of our report are as follows. This was a retrospective review of HIV patients, including all of patients participating in treatment of HIV/AIDS in the outpatient clinic of NHTD, in the period from 2005 to 2011. This subpopulation of the clinic is not representative of all HIV patients in the clinic or all HIV patients in Vietnam. Nonetheless, our results are consistent with other studies and are relevant for improving the care of HIV/AIDS patients.

## 5. Conclusion

Coinfection with viral hepatitis is common in HIV patients. Therefore, reducing parenteral transmission of HCV and HBV requires that current prevention messages be revised to alert HIV person to the relevant risks factors, including emphasis on the importance of reducing or eliminating all equipment-sharing practices and safe sexual activity. A good target for a targeted intervention strategy to prevent hepatitis B, hepatitis C, and HIV coinfection could be based on family and community. Consideration should be paid to integrating hepatitis B vaccination for IDUs into large-scale HIV prevention programs. All HIV-infected persons should be supported in a program of routine HBV and HCV screening testing. We should continue to find out more about the impact and evolution of coinfection.

## Figures and Tables

**Table 1:** Demographic, risk behaviors, and clinical stage at baseline of the studied population (*n* = 724).

	Overall sample *N* (%)	HBV infection		HCV infection		HBV/HCV infection	
		Number of pos (%)	*P*	Number of pos (%)	*P*	Number of pos (%)	*P*
Demographic							
Age group (years)							
<30	168 (23.2)	11 (6.5)	0.70	32 (19.0)	<0.001	10 (6.0)	0.23
30–39	375 (51.8)	32 (8.5)		189 (50.4)		30 (8.0)	
40–49	116 (16.0)	11 (9.5)		24 (20.7)		6 (5.2)	
≥50	65 (9.0)	7 (10.8)		11 (16.9)		1 (1.5)	
Sex							
Male	465 (64.2)	34 (7.3)	0.15	234 (50.3)	<0.001	44 (9.5)	<0.001
Female	259 (35.8)	27 (10.4)		22 (8.5)		3 (1.2)	
Marital status							
Never married	98 (13.5)	45 (45.5)	<0.001	69 (70.4)	<0.001	10 (10.2)	<0.04
Currently married	543 (75.0)	14 (2.6)		182 (33.5)		36 (6.6)	
Divorced/widowed	83 (11.5)	2 (2.4)		5 (6.0)		1 (1.2)	
Geographic Domicile							
Hanoi	296 (40.9)	18 (6.1)	0.06	91 (30.7)	0.13	16 (5.4)	0.32
Other provinces	428 (59.1)	43 (10.0)		155 (36.2)		31 (7.2)	
Risk factor associated							
Injection drug used	443 (61.2)	18 (4.0)	<0.001	195 (44.0)	<0.001	24 (5.0)	<0.001
Sexual intercourse	165 (22.8)	43 (26.0)		46 (27.9)		5 (3.0)	
Mixed	116 (16.0)	0 (0)		15 (11.2)		18 (15.5)	
Clinical stage classification							
Stage 1	262 (27.6)	23 (8.8)	0.27	47 (17.9)	<0.001	14 (5.3)	<0.001
Stage 2	132 (18.2)	16 (12.1)		63 (47.7)		9 (6.8)	
Stage 3	74 (10.2)	6 (8.1)		31 (41.9)		12 (16.2)	
Stage 4	256 (35.4)	16 (6.2)		115 (44.9)		12 (4.7)	
